# The Institutional Treatment of Venereal Disease

**Published:** 1916-02-26

**Authors:** 


### THE INSTITUTIONAL TREATMENT OF VENEREAL DISEASE.

### Isolation and Other Knotty Problems.

It is an open secret in public health circles that, had it not been for the war, preparations would shortly have been in hand for dealing with the treatment of venereal disease in this country. Indeed it was mooted, we believe, that the machinery now fairly well organised in the majority of districts and towns for attacking the tuberculosis problem would be utilised directly or indirectly for this purpose. We have on previous occasions dealt with some of the aspects of this great problem, particularly in connection with the Report by Dr. R. W. Johnstone, of the Local Government Board, which was published two years ago, and in an article describing the system of controlled treatment and notification in force in Denmark.

### Institutional Accommodation.

Before any considerable pressure can be exerted in the direction of compelling victims of this class of disease to submit to adequate and complete treatment, it is obvious that access to the sources of such treatment must be made readily available. With the provision of the "out-patient" order of treatment it is not proposed to deal at the moment, but attention may be called to the vexed question of institutional treatment of venereal disease. Shall these patients be treated only in special hospitals and kept apart from other patients, or shall they be eligible for admission into any hospital? The question is a difficult one. To distinguish between venereal and non-venereal disease is to the medical mind primarily invidious, but the admission into general wards of subjects of the former class of diseases is a principle not likely readily to be accepted by governing bodies of voluntary hospitals. Not unnaturally, other patients in the wards might raise objections, even if there were no risks of infection and apart from moral influences. It goes without saying that really infectious or undesirable patients would have to be accommodated in small isolation wards, but the advocates of this "mixed" policy do not desire that such small wards should be devoted entirely to venereal patients and thus become branded; they should be open to sufferers from other infectious diseases. It may be supposed that patients having venereal disease are loth to enter a special hospital or a special ward because they fear that the nature of their complaint will be disclosed; but in actual practice such a disclosure would, we suspect, not long be delayed in a "general" ward. Adoption by a general hospital of this "mixed" policy is, again, not unlikely to cause the institution to fall into disfavour and eventually to suffer financially.

In our largest towns special hospitals will almost certainly be found the more practicable and the more convenient: a sufficiently large number of patients will be available to make a special institution worth while, and a specialist staff could be exclusively retained. But in a great many towns the "mixed" policy will have to be carried out, and we believe that, given a fair and judicious trial, it will be shown that most of the difficulties referred to above are capable of being overcome. In course of time the conception of venereal disease as a retribution for sin will become less prevalent than it is, unfortunately, at the present time, and thereby the greatest hindrance of all to proper treatment and the safeguarding of the innocent will be removed. What effect the aftermath of the war will have on the attitude of the "retributionists" it is interesting to speculate. It is permissible to assume that the injustice of their standpoint will penetrate the minds of the majority.

